# Morphological and karyotypic differences within and among populations of *Radopholus similis*

**DOI:** 10.3897/zookeys.444.8186

**Published:** 2014-10-07

**Authors:** Chun-Ling Xu, Yun Li, Hui Xie, Xin Huang, Wen-Jia Wu, Lu Yu, Dong-Wei Wang

**Affiliations:** 1 Laboratory of Plant Nematology /Research Center of Nematodes of Plant Quarantine/Province Key Laboratory of Microbial Signals and Disease Control, College of Natural Resources and Environment, South China Agricultural University, Wushan Street, Guangzhou, Guangdong Province, China

**Keywords:** Burrowing nematode, optical and SEM microscopy, morphological comparison, karyotype

## Abstract

Twenty populations of *Radopholus similis* from three countries and different hosts (19 populations from ornamental plants and one population from ginger) were compared using morphological characters, morphometrics and karyotype between progeny from both single females and 30 females of each population. Morphological diversity existed in and among the populations, even within the progeny nematodes from single nematodes compared to that of 30 females. The labial disc shape, the number of head annuli, the terminated position of lateral lips, the number of genital papillae before cloacal apertures and female and male tail terminal shape showed variation. In addition, genital papillae arranged in a double row before cloacal apertures was first found in two ornamental populations. The karyotype of all the 20 populations was n = 5. Combining our results and previous studies, we support that *Radopholus citrophilus* is a synonym of *Radopholus similis*, and that it is not possible to distinguish physiological races or pathotypes of *Radopholus similis* according to morphological characters or karyotype.

## Introduction

The burrowing nematode, *Radopholus similis* (Cobb, 1893) Thorne, 1949, is an important parasitic plant nematode has made great damages on many economic crops, and is on the list of quarantined pests in many countries (Anonymous 2004; Haegeman et al 2010). *Radopholus similis* is widely distributed in tropical and subtropical regions with extensive host ranges, up to 250 species (Holdeman 1986), including not only *Musa* spp., *Citrus* spp., *Piper nigrum*, *Saccharum sinensis*, *Camellia sinensis* and other economic crops, but also ornamental plants belonging to the Araceae, Marantaceae, Bromeliaceae, Musaceae and Palmae (Williams and Siddiqi 1973, Bridge 1993, Duncan and Cohn 1990; Loof 1991, Anonymous 2004).

In the mid-1960s, Ducharme and Birchfield (1956) proposed that there were two physiological races (pathotypes) of *Radopholus similis*; the morphologically similar banana race and the citrus race. The banana race infected only banana but not citrus and vice versa (Ducharme and Birchfield 1956, Hahn et al. 1996b, Valette et al. 1998). Van Weerdt (1958) measured the morphological characters of these two races, and did not find any differences between the two. Huettel and Yaegashi (1988) reported that there were some differences in the morphology of the female labial disc, the lateral lip position, the number of annuli terminated at the vulva and the number of genital papillae anterior to the cloacal aperture in the male between the two races when viewed by scanning electron microscope (SEM). These morphological differences were thought to be used to differentiate between the different races; therefore, Huettel et al. (1984) proposed to treat the citrus race as an independent species, *Radopholus citrophilus* Huettel, Dickson, David & Kaplan, 1984. However, Valette et al. (1998) did not agree, studying two banana races of *Radopholus similis* from Africa by SEM, and finding that there were morphological overlaps within the four proposed distinguishing characters, and thereby treated *Radopholus citrophilus* as a synonym of *Radopholus similis*. These findings were later confirmed by other studies (Koshy et al. 1991, Elbadri et al. 1998, Elbadri et al. 1999a, 1999b).

Cytogenetics is an important tool to reveal the phylogenetic relationships within nematode species (Triantaphyllou 1970), and the differences in karyotype is of phylogenetic significance in the study of indistinguishable races within species (Triantaphyllou and Hirschmann 1966). Karyotype and cell development have been reported in the study of many parasitic plant nematodes, i.e. root-knot nematodes, *Meloidogyne* spp., pine wood nematodes, *Bursaphelenchus* spp., and the burrowing nematodes *Radopholus* spp. Some nematologists thought chromosome number, egg cells and gonad cells were helpful in distinguishing different populations (Huettel and Dickson 1981a, Huettel et al. 1984a, 1984b, Aoyagi and Ishibashi 1983, Bolla and Boschert 1993, van der Beek et al. 1998, Kaplan and Opperman 2000, Hasegawa et al. 2004, Liu and Williamson 2006). Huettel and Dickson (1981a, b) even reported that the haploid chromosome number of karyotype of two physiological races of *Radopholus similis* was n = 4 (banana race) and n = 5 (citrus race). Huettel et al. (1984b) confirmed the result by studying 17 populations of *Radopholus similis*, proposing that using haploid chromosome number of karyotype was more reliable to distinguish citrus races from banana races. However, Rivas and Roman (1985) and Hahn et al. (1996a) found that the haploid chromosome number of some banana races was also 5. While Goo and Sipes (1999) studied the haploid chromosome of six isolates of *Radopholus similis* collected from Anthurium, banana and Calathea in Hawaii, and found the chromosome number ranged within isolates from n = 4–7. Kaplan and Opperman (2000) studied the karyotype of 56 populations of *Radopholus similis*, and demonstrated that citrus races and banana races could mate and produce offspring showing a similar morphology of *Radopholus similis*, and all the chromosome numbers of these offspring was 5; therefore, it was inappropriate to determine different races only according to different karyotypes.

In this study, morphometry, ultrastructural morphology and haploid karyotype of the progeny of single females and 30 females from the same population of 20 populations of *Radopholus similis*, collected from greenhouses and plants intercepted from abroad, were studied and analyzed.

## Materials and methods

### Nematode populations

Twenty populations of *Radopholus similis* were established on carrot disc cultures (Moody et al. 1973). The populations were originally isolated from ornamental plants and ginger (Table 1). Sterile water was added into the carrot disc dishes to get nematodes suspension in the benchtop, and a single young female was picked and inoculate to a new carrot disc callus with a tiny sterile water drop on it. At the same time, 30 females from the same population weretransferred to another new callus in the benchtop. Progeny from 30 females and single females of each *Radopholus similis* population were collected for further study after 60 days on carrot discs.

**Table 1.** Origin of *Radopholus similis* populations used in this study.

Code	Original collection locations	Host plant
RsA	Netherlands (intercepted)	*Calathea zebrina*
RsB	China	*Ravenea rivularis*
RsC	Netherlands (intercepted)	*Calathea* sp.
RsD	China	*Chamaedorea cataractarum*
RsE	China	*Philodendron* sp.
RsG	China	*Chamaedorea cataractarum*
RsH	China	*Philodendron* sp.
RsI	China	*Anthurium andraeanum*
RsJ	China	*Anthurium andraeanum*
RsK	China	*Calathea zebrina*
RsL	China	*Epipremnum aureum*
RsM	Malaysia (intercepted)	*Stranvaesia* sp.
RsN	China	*Chrysalidocarpus lutescens*
RsP	China	*Calathea zebrina*
RsT	China	*Calathea* sp.
RsS	China	*Calathea makoyana*
RsV	China (Hong Kong)	*Anthurium andraeanum*
RsW	China	*Anthurium andraeanum*
RsY	China	*Anthurium andraeanum*
RsXj	Singapore (intercepted)	*Zingiber officinale* Roscoe

**Morphological study.** Specimens were heat-killed and fixed by adding 4% hot formaldehyde, and transferred to anhydrous glycerin according to Seinhorst’s method (Seinhorst 1959). Females and males were separated and mounted on permanent slides (Seinhorst 1959), and 20 females and 20 males were measured for progeny of single female and 30 females of each population, respectively. All measurements and photomicrographs were made using a Nikon 90i microscope with camera. For ultrastructure morphological observations, the method described by Xu et al. (2009) was utilized, and the parameters were measured according to de Man’s formula (de Man 1890). All the progeny of single females were coded by adding “s” behind the population code number, e.g. RsA represented the progeny of 30 females of *Calathea zebrina* population inoculated on carrot discs, and RsAs represented the progeny of single female inoculated on carrot discs of the same population.

**Karyotypic study.** In order to observe choromosomes in eggs or adult nematodes of *Radopholus similis*, fluorescence staining method was used as described by Kaplan and Opperman (2000) only with minor modifications. In brief, nematodes and eggs were collected from the carrot disc dishes and washed twice with sterile distilled water. After the supernatant was removed, 200 μl of Carnoy’s solution was added to fix the pellet for 5 minutes. After removal of the fixative, the pellet was incubated in 100% methanol for 20 minutes. The pellet was rinsed twice with phosphate buffered saline (PBS) for 5 minutes, then incubated in a washing buffer for 10 minutes and washed again with PBS and sterile water. The nematodes and eggs were stained with DAPI (4’, 6-diamidino-2-phenylindole) (Sigma-Aldrich Inc.) (1 μg/ml) for 5 minutes, washed once with PBS, and incubated overnight in fluorescence quenching agent. The specimens were made in half permanent slides, and viewed with a Nikon fluorescent microscope (90i).

## Results

### Morphological characteristics

**Figure 1.**
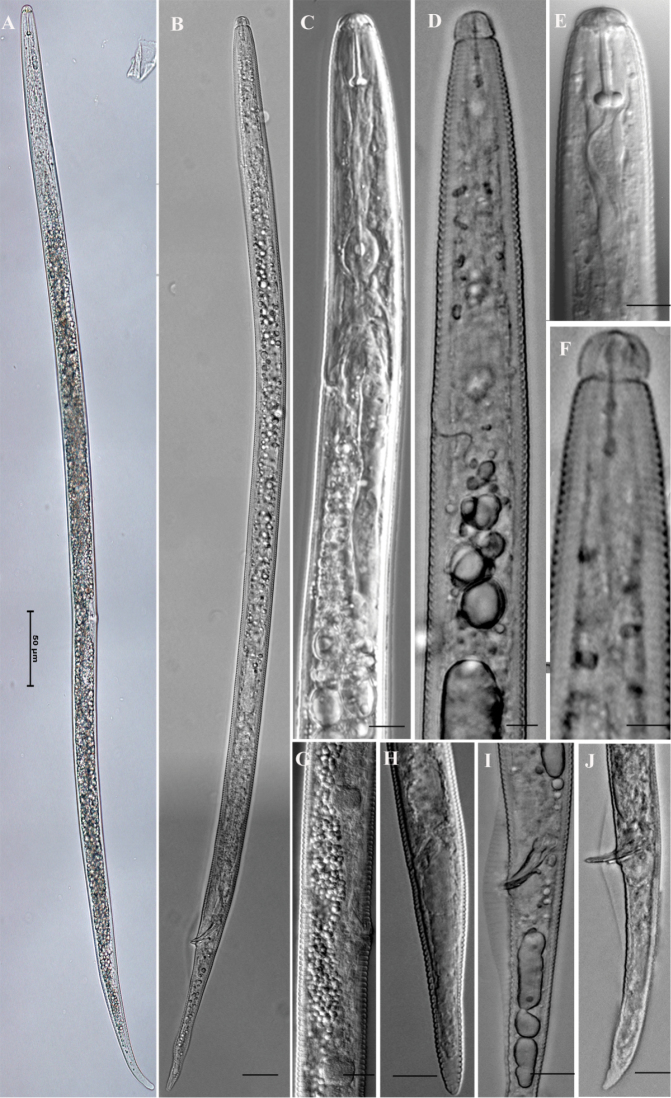
Morphology of *Radopholus similis* Female: **A** whole body **C** anterior part of body **E** lip region and stylet **G** vulva region **H** tail. Male: **B** whole body **D** anterior part of body **F** lip region and stylet **I** cloacal region **J** tail Scale bar: **A** = 50 µm; **B, H** = 20 µm; **C, E, G, I, J** = 10 µm; **D, F** = 5 µm.

**Figure 2.**
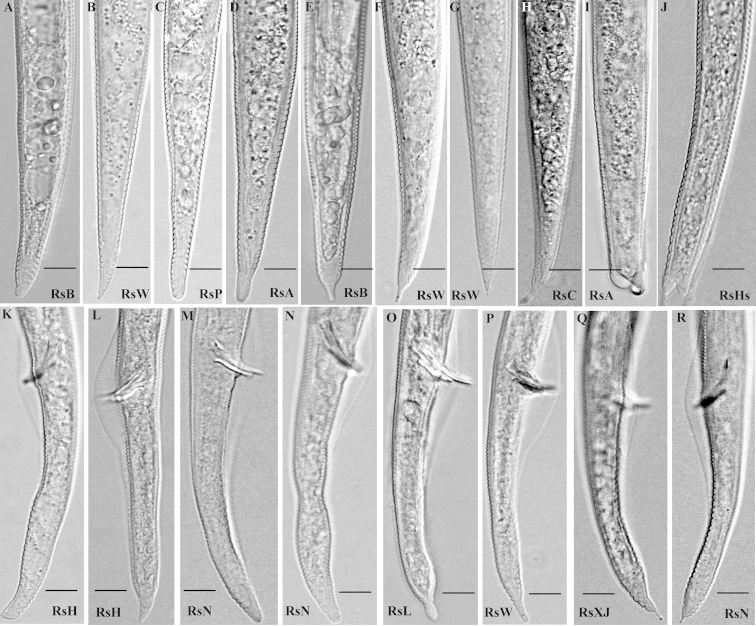
Tail morphology of *Radopholus similis* Female: **A–B** TypeItail **C–D** Type II tail **E–H** Type III tail **I–J** Type IV tail. Male: **L–M** TypeItail **K, N** Type II tail **O–R** Type III tail. **A, E:** RsB; **B, F, G, P:** RsW; **C:** RsP; **D, I:** RsA; **H:** RsC; **J:** RsHs; **K–L:** RsH; **M–N:** R: RsN; **O:** RsL; **Q:** RsXJ. Scale bar: **A–R** = 10 µm.

All 20 populations of *Radopholus similis* exhibited all of reported morphological characters (Tables 2, 3). The female body was almost straight to slightly ventrally curved after heat killed (Figure 1A). The head was low and a little rounded, continuous or slightly offset with body contour (Figure 1E, Figure 5A–C). Lateral field had four incisures and obviously areolated (Figure 4G–H). The middle band was equal or a little wider than the two lateral bands. The stylet was well-developed, shape and size of dorsal basal knobs and two subventral knobs almost identical in shape and size; dorsal gland orifice was near the stylet base (Figure 1E). The excretory pore opened ventrally 0–2 annuli behind the hemizonid, approx. 2–3 annuli long in diam. The oesophageal gland overlapped the intestine dorsally (Figure 1C). The vulva situated in the postmedian part of the body. The vulva was flat or slightly projecting (Figure 1G). The reproductive system was didelphic, extended, with oocytes in a single row. The spermatheca was round or oval, with rod-shaped sperm (Figure 1G). The gonad inflexion exists in some populations and the anterior gonad was longer than the posterior one. The tail was mostly subconoid (Figure 1H, Figure 2A–I), longer than 70 μm, with average hyaline part of tail longer than 5.6 μm. Male: The lip region was high and round, hemispherical, clearly offset with body contour, bearing 3–5 annuli (Figure 1F, Figure 5D–F). The stylet was weak, without base knob or only with slightly expanded base. The median bulb and gland of the esophagus degenerate (Figure 1D). The excretory pore opened ventrally at 0–1 annulus behind hemizonid. Single testis extended forward. The gubernaculum extended over cloacal pore, approx. half length of spicule. The bursa wasobvious, extending more than 47%–90% length of tail (Figure 1I–J, Figure 5R–V).

**Table 2.** Morphometrics of *Radopholus similis* females from offspring of mixed females and single female from 20 populations (n = 20), respectively (measurements: µm).

Code^a^	*L*	*a*	*b*	*b*’	*c*	*c*’	*V*	*V*’	*G_1_*
RsA	781.6±31.48 (700–842.5)	27.9±1.61 (24.4–30.6)	6±0.35 (5.3–6.6)	4.9±0.29 (4.3–5.3)	9.1±0.52 (8.4–10.5)	4.4±0.28 (3.9–4.9)	55.1±1.2 (52.4–56.7)	62.2±1.25 (59.4–64.3)	28.7±3.21 (20.6–33.4)
RsAs	743.4±26.54 (705–798.8)	35.1±2.2 (31.6–39)	6±0.34 (5.5–6.9)	4.8±0.19 (4.4–5.1)	8.7±0.37 (8.1–9.7)	5.6±0.38 (4.8–6.4)	55.5±0.83 (53.2–57.7)	62.8±0.9 (60.2–64.8)	20.3±2.22 (18.1–26.7)
RsK	706.8±52.57 (513.8–776.3)	26.2±2.2 (17.9–28.7)	6.1±0.54 (4.8–7)	4.7±0.37 (3.3–5)	8.4±0.68 (5.7–8.9)	4.3±0.3 (3.7–4.9)	57.9±6.13 (52.4–83)	64.1±1.78 (60–68.6)	35.0±5.25 (25.3–47.2)
RsKs	682.4±41.81 (617.5–755)	31.5±2.58 (26.3–36.1)	5.6±0.47 (5–6.9)	4.5±0.3 (3.7–5.1)	8.2±0.39 (7.5–9)	5.2±0.59 (4.2–6.4)	56±0.92 (53.1–58.7)	63.7±1.17 (60–66)	23.1±3.3 (19.5–32.5)
RsP	674.8±33.08 (597.5–748)	31.4±1.81 (28.1–34.2)	5.5±0.3 (4.9–6)	4.3±0.18 (4–4.6)	9.2±0.51 (7.7–9.9)	4.3±0.36 (3.6–4.9)	56.9±1.82 (55.3–64.2)	63.4±0.69 (62.1–64.9)	23.5±2.79 (19–30.2)
RsPs	667.7±21.87 (613–713.8)	33.1±1.46 (30.1–36.4)	5.4±0.4 (4.5–6.1)	4.1±0.19 (3.8–4.5)	9.1±0.39 (8.3–9.7)	4.5±0.32 (4–5.1)	56.8±1.55 (53.2–62.4)	63.9±1.81 (59.8–70.2)	22.9±2.36 (19.9–29.9)
RsC	795.9±27.64 (730–843.8)	27.1±2.3 (22.1–29.4)	5.8±0.58 (4.9–6.7)	4.8±0.18 (4.5–5.1)	8.8±0.42 (7.9–9.6)	4.6±0.32 (4.1–5.3)	55.2±0.91 (53.2–57.1)	62.3±1.06 (60.2–65.1)	33.6±2.49 (30.1–37.7)
RsCs	722.3±34.64 (647.5–767.5)	33.6±2.79 (28.5–37.9)	5.8±0.42 (4.8–6.6)	4.6±0.23 (4.1–5.2)	8.8±0.31 (8.2–9.4)	5.2±0.47 (4.5–6.4)	55.1±1.82 (49.1–57.9)	62.4±1.77 (55.6–65.5)	23.6±3.56 (18.4–30.9)
RsS	743.8±23.6 (702.5–778.8)	28±1.47 (25.5–30.6)	6.3±0.7 (5.3–7.9)	4.9±0.16 (4.5–5.1)	8.5±0.34 (8–9.3)	4.8±0.32 (4.1–5.7)	55.3±1.55 (51.9–60.2)	62.7±1.8 (58.3–68.4)	27.7±2.53 (23.2–31.5)
RsSs	719.9±29.35 (670–778)	30.8±3.04 (26.2–36.6)	5.9±1.59 (5–14.2)	4.5±0.22 (4.1–4.9)	8.6±0.44 (7.9–9.7)	5±0.52 (3.9–6.1)	56.4±1.89 (54.1–61.6)	63.8±2.12 (61.2–70.3)	23±3.24 (18.6–29.9)
RsT	736.9±22.67 (701.3–775)	29.6±1.96 (26.9–33.7)	5.3±0.32 (4.7–6.1)	4.4±0.19 (4.1–4.8)	9.3±0.52 (8.4–10.6)	4±0.33 (3.4–4.5)	56.2±0.92 (54.3–57.9)	63.1±0.97 (61.2–65.1)	22.9±1.59 (20–26.6)
RsTs	664.8±26.97 (610–718.8)	33.4±2.69 (28.2–39.1)	5.4±0.29 (4.7–5.9)	4.1±0.22 (3.7–4.5)	9.3±0.41 (8.3–10)	4.6±0.31 (3.9–5.1)	57.3±1.44 (51.2–59.4)	64.2±1.46 (58–66.4)	20.9±2.49 (18.2–28.5)
RsB	678.5±29.83 (625–742.5)	27.9±1.34 (25.5–30.5)	5.1±0.35 (4.5–5.6)	4.3±0.18 (4–4.6)	8.8±0.48 (8.2–9.8)	4±0.24 (3.6–4.6)	56.4±0.81 (54.7–57.7)	63.7±0.99 (61.7–65.3)	23.7±2.87 (19.5–31.4)
RsBs	677.9±34.8 (608.8–743.8)	31.1±1.73 (27.8–34.9)	5.4±0.33 (4.8–6.2)	4.2±0.2 (3.8–4.6)	9.1±0.36 (8.5–9.8)	4.4±0.36 (3.9–5.5)	56.8±1.24 (54.2–61)	63.6±1.37 (61–68.6)	22.3±2.56 (19.1–27.4)
RsD	735.4±30.88 (687.5–785)	28±1.66 (25.2–31.1)	5.9±0.41 (5.4–6.9)	4.8±0.24 (4.3–5.1)	9.6±0.44 (8.9–10.3)	3.7±0.25 (3.4–4.4)	57.1±0.71 (55.4–58.3)	63.8±0.91 (62.2–65.7)	27.8±2.62 (23.8–31.4)
RsDs	651.6±16.06 (617.5–692.5)	32.9±2.41 (28.7–37.6)	5.5±0.24 (5–6)	4.4±0.19 (3.9–4.7)	9.5±0.43 (8.6–10.4)	4.3±0.31 (3.8–5.1)	57.4±1.29 (54.3–62)	64.2±1.41 (60.9–69.4)	20.7±1.55 (18.1–25.5)
RsG	741.1±30.95 (681.3–810)	28.9±1.75 (25.8–32.4)	5.6±0.27 (5.2–6)	4.5±0.24 (4.1–5)	9.1±0.37 (8.4–9.8)	4.1±0.19 (3.6–4.4)	56.2±0.81 (54.7–57.5)	63.2±0.97 (61.6–64.9)	26.6±3.72 (21.7–35.6)
RsGs	661.6±24.14 (606.3–713.8)	34.6±2.18 (28.6–38.8)	5.6±0.37 (4.9–6.5)	4.1±0.18 (3.8–4.5)	9.2±0.32 (8.5–9.9)	4.6±0.32 (4–5.3)	56.5±0.84 (55.2–59)	63.5±0.92 (62.2–66.7)	20.9±1.95 (16.8–25.6)
RsE	676.9±29.04 (612.5–727.5)	28.9±1.91 (25.7–32.3)	5.2±0.25 (4.7–5.6)	4.4±0.16 (4–4.6)	8.9±0.48 (8.2–9.8)	4.1±0.31 (3.5–4.7)	56.7±0.81 (54.5–58.3)	63.9±0.95 (61.1–65.5)	23.7±2.91 (19.4–31.5)
RsEs	661.9±50.81 (545–747.5)	33.1±2.76 (27.4–37.3)	5.4±0.39 (4.6–6.2)	4.2±0.28 (3.5–4.7)	9.2±0.44 (8.5–10.3)	4.6±0.45 (3.9–5.6)	57±1.06 (54.9–59.7)	64±1.22 (61.3–67.3)	22.2±2.96 (16.8–29.2)
RsH	764.1±27.22 (705–803.8)	30±2.9 (25.6–35.7)	6±0.53 (5.4–7.1)	4.9±0.21 (4.4–5.4)	8.8±0.34 (8.2–9.4)	4.7±0.33 (4.2–5.1)	55.9±1.08 (53.5–58.2)	63.4±1.48 (60.6–67.9)	28±2.73 (22.8–33.3)
RsHs	682.9±32.03 (622.5–743.8)	32.7±1.97 (28.6–37)	5.7±0.33 (4.9–6.4)	4.4±0.32 (3.8–4.9)	8.4±0.27 (7.9–9.1)	5.6±0.34 (5–6.6)	56.1±2.04 (53.9–61.6)	63.6±2.32 (60.9–70)	19.3±1.6 (16.4–22.7)
RsI	680.8±21.62 (643.8–730)	29.9±1.88 (26.7–34.6)	5.7±0.49 (5–6.8)	4.5±0.19 (4.1–4.9)	9.6±0.58 (8.7–11.2)	4±0.32 (3.4–4.5)	56.7±1.08 (54.6–58.7)	63.3±1.06 (61.1–65.1)	23.6±3.61 (18.7–31.6)
RsIs	666.6±25.04 (611.3–725)	32.7±2.16 (26.7–35.2)	5.5±0.28 (5–6.1)	4.4±0.19 (3.9–4.8)	9.5±0.55 (8.6–11.1)	4.5±0.42 (3.1–5)	57.4±1.68 (56–64.2)	64.1±1.32 (62.4–68.3)	21.8±3.4 (18–30.2)
RsJ	718.2±18.03 (682.5–742.5)	26.5±1.22 (24.8–29.6)	5.8±0.4 (5.1–6.9)	4.9±0.15 (4.6–5.1)	8.6±0.46 (8–9.5)	4.2±0.18 (3.9–4.5)	57.3±1.81 (55–63.8)	64.9±2.05 (62.4–72.2)	31.5±2.47 (27.4–35.8)
RsJs	661.2±25.38 (617–718.8)	32.1±2.77 (24.7–37.1)	5.6±0.32 (5.1–6.4)	4.5±0.23 (4–4.9)	8.1±0.33 (7.5–8.8)	5.2±0.39 (4.1–5.8)	56.5±1.19 (54.6–60.4)	64.3±1.17 (62.1–67.4)	21.5±2.24 (18.8–28.4)
RsV	730.5±29.23 (675–775)	30.3±1.7 (27–33.9)	5.3±0.26 (4.9–6)	4.4±0.19 (4–4.8)	9.3±0.56 (8.5–10.2)	4.1±0.33 (3.6–4.7)	56.4±0.81 (54.9–57.5)	63.3±0.83 (61.7–64.5)	27.7±2.96 (22.4–32.8)
RsVs	693.8±41.54 (632.8–797.5)	32.1±1.69 (29.3–35.1)	5.5±0.4 (4.9–6.9)	4.3±0.24 (3.9–5)	9.2±0.44 (8.4–10.5)	4.5±0.4 (3.7–5.2)	57.3±1.3 (54.8–60.8)	64.4±1.4 (61.9–67.7)	22.3±3.62 (17.7–31.5)
RsW	742.7±20.94 (717.5–790)	26.7±0.95 (24–28)	6.1±0.28 (5.8–6.8)	5.1±0.26 (4.3–5.5)	8.6±0.38 (7.8–9.3)	4.3±0.33 (3.4–4.8)	56.4±1.18 (54.7–59.7)	64±1.27 (61.7–67.8)	31.5±4.39 (24.4–40.3)
RsWs	659.3±26.3 (617.5–731.3)	29.3±2.6 (24.9–33.9)	5.6±0.45 (5–6.6)	4.5±0.35 (3.9–5.2)	8.2±0.37 (7.7–9.2)	5±0.48 (4.3–5.8)	56±0.91 (54.4–58.2)	63.7±1.21 (60.3–65.5)	23.7±3.06 (19.3–28.9)
RsY	731.7±19.73 (690–756.8)	26.5±1.3 (24.3–28.2)	6.2±0.35 (5.8–6.8)	4.7±0.2 (4.5–5.1)	9.5±0.43 (8.9–10.1)	3.7±0.12 (3.6–4)	57.1±0.79 (56–58.5)	63.8±0.89 (62.1–65.4)	29±4.79 (19.1–34.5)
RsYs	689.7±24.91 (628.8–732.5)	32.9±1.82 (29.6–38)	5.5±0.32 (4.9–6.2)	4.3±0.14 (4–4.6)	9.2±0.34 (8.8–10)	4.6±0.29 (4.1–5)	57.1±0.96 (55.5–59.1)	64.1±1.09 (62.2–66.4)	20.9±1.88 (18.3–26.1)
RsL	665.8±45.12 (615.5–812.5)	31.7±1.89 (28.8–36.1)	5.4±0.44 (5–6.8)	4.1±0.26 (3.7–4.7)	9.1±0.34 (8.6–9.9)	4.5±0.31 (3.9–4.8)	57.4±0.95 (55.5–58.8)	64.4±0.9 (62.5–66)	21.7±1.6 (20–25.1)
RsLs	661.8±33.1 (597.5–742)	31±1.86 (27.1–34.7)	5.5±0.26 (5–6)	4.3±0.19 (3.8–4.6)	9.2±0.5 (8.4–10.4)	4.2±0.26 (3.7–4.6)	56.4±1.27 (54.1–60.6)	63.3±1.52 (61.1–67.9)	22.1±2.23 (19.5–30.1)
RsM	656.7±21.99 (595–688.8)	30.8±1.29 (28.8–33.7)	5.1±0.31 (4.6–5.9)	4.3±0.23 (3.9–4.7)	9.2±0.42 (8.1–10.2)	4.3±0.28 (3.7–5)	56.4±1.16 (52.9–58.2)	63.2±1.52 (59.1–65.8)	20.1±1.2 (17.8–22.8)
RsMs	666.9±32.64 (613.8–788.8)	33.8±1.78 (28.8–37.2)	5.5±0.33 (4.9–6.2)	4.3±0.2 (3.9–4.7)	9.3±0.46 (8.2–10)	4.5±0.39 (3.8–5.4)	57±0.75 (55.7–59.3)	63.9±0.89 (62.1–66.5)	20.7±1.49 (17.1–24.4)
RsN	689.3±30.12 (630–735)	30.8±1.77 (25.9–33.5)	5.3±0.26 (4.8–5.9)	4.4±0.17 (4.2–4.7)	9±0.47 (8.2–10.2)	4.5±0.38 (3.6–5.3)	56.3±1.03 (54.5–58.7)	63.4±1.34 (60.5–66.1)	24.2±2.14 (19.7–28.7)
RsNs	666.3±19.02 (632.5–705)	33.3±2.36 (29–37.6)	5.4±0.22 (4.9–5.8)	4.2±0.17 (3.9–4.5)	9.1±0.36 (8.4–9.9)	4.6±0.29 (3.7–5)	57.2±0.86 (55.8–58.7)	64.4±0.95 (62.4–66)	20.4±2.36 (17.6–27.8)
RsXJ	708.3±21.11 (671.3–756.3)	26.5±1.38 (24.4–28.9)	5.3±0.2 (4.9–5.7)	4.8±0.13 (4.5–5)	8.4±0.33 (7.5–8.9)	4.2±0.22 (3.8–4.5)	56.7±1.45 (54.2–61.1)	64.4±1.59 (61.6–69.3)	30.4±3.66 (26.3–40.3)
RsXjs	659.9±16.65 (631.3–691.3)	29.9±1.08 (27.6–32.6)	5.7±0.3 (5.2–6.4)	4.6±0.2 (4.2–5.1)	8.5±0.37 (7.9–9.5)	4.9±0.27 (4.3–5.3)	56.8±1.31 (55.1–61)	64.2±1.76 (58.5–69.2)	21.1±1.31 (17.5–23.3)

Code	*G_2_*	*Stylet*	*Tail*	*h*	*Ran*	*MB*	Distance from stylet knob to dorsal gland origin	Pharynx length
RsA	25.5±2.93 (20.6–30)	18.5±0.71 (17.3–19.4)	85.9±5.28 (77.5–95)	11.9±1.84 (8.7–15.3)	47.1±4.23 (40–54)	63.7±2.35 (59.2–67.8)	4.6±0.52 (4.1–5.1)	144.2±9.8 (131.6–170.3)
RsAs	18.7±1.65 (16.3–22.7)	19.1±0.44 (18.4–19.4)	85.3±4.05 (77.5–95)	10.5±2.6 (7.1–18.9)	50.3±4.09 (44–57)	63.8±2.59 (59.2–69.4)	4.6±0.48 (4.1–5.1)	138±7.46 (123.4–154)
RsK	31.7±5.79 (22.6–47.7)	17.8±0.62 (17.3–19.4)	84.4±2.97 (78.8–90)	9.6±2.06 (6.1–14.3)	43.4±4.29 (38–55)	62.9±2.69 (57.1–69.4)	4.5±0.5 (4.1–5.1)	134.1±4.79 (123.4–139.7)
RsKs	21.1±1.96 (18.2–25.6)	18.5±0.47 (17.3–19.4)	83.3±4.12 (76.3–90.5)	9.5±1.75 (6.1–13.5)	44.1±3.13 (38–52)	60.2±2 (56.1–64.3)	4.3±0.41 (3.1–5.1)	132.2±8.15 (114.2–153)
RsP	22.7±2.26 (20–28.6)	19.1±0.45 (18.4–19.4)	73.4±4.52 (66.3–82.5)	11.2±1.01 (9.2–13.3)	50.9±6.96 (32–61)	60.6±2.08 (55.1–64.3)	4.4±0.53 (3.1–5.1)	140±6.36 (130.6–151)
RsPs	21.3±1.56 (17.9–24.7)	19.1±0.43 (18.4–19.4)	73.8±4.22 (67–83.8)	11.6±1.17 (9.2–14.1)	51.8±5.37 (38–60)	62.1±1.91 (58.1–66.3)	4.1±0.26 (4.1–5.1)	143.9±7.94 (126.5–159.1)
RsC	28.9±2.21 (24.9–32.4)	18.7±0.67 (17.3–19.4)	90.9±4.18 (85–102.5)	12.3±2.13 (9.2–17.3)	45.4±3.79 (39–52)	65.8±1.67 (63.2–69.9)	5±0.4 (4.1–5.6)	144.6±6.72 (133.6–160.1)
RsCs	20.5±3.01 (16.3–28.3)	19±0.51 (17.9–19.4)	82.4±4.81 (72.5–92.5)	9.7±1.49 (7.1–13.8)	44.4±4.84 (36–57)	64.6±2.35 (60.2–70.4)	4.3±0.42 (4.1–5.1)	139.2±7.36 (125–154)
RsS	25.9±2.79 (20.4–30)	18.5±0.6 (17.3–19.4)	87.5±3.79 (80–93.8)	11±2.37 (8.2–15.3)	46.1±3.54 (40–51)	63.9±1.63 (61.2–66.3)	4.3±0.41 (3.6–5.1)	133.6±7.59 (124.4–158.1)
RsSs	20.7±2.28 (17.1–26.6)	19.1±0.42 (18.4–19.9)	83.9±5.79 (72.5–93.8)	9.4±2.38 (2.6–13.3)	48.8±5.37 (39–59)	64.8±2.68 (60.2–70.4)	4.2±0.33 (4.1–5.1)	141.7±8 (124.4–154)
RsT	21.7±3.19 (18.4–30.7)	18.8±0.51 (18.4–19.4)	79.3±3.88 (72. 5–86.3)	12.8±1.28 (10.7–15.8)	47.7±4.88 (37–58)	63±1.67 (60.2–66.3)	4.2±0.31 (4.1–5.1)	147.7±7.26 (134.1–163.2)
RsTs	19.6±1.72 (17.4–24.9)	19±0.49 (18.4–19.9)	71.8±3.57 (63–78.8)	13±1.04 (11.2–14.8)	45.8±3.46 (39–53)	63.4±2.67 (57.1–67.3)	4.2±0.39 (3.1–5.1)	143.7±6.91 (131.6–164.2)
RsB	22.2±1.6 (19.5–24.)	18.1±0.47 (17.3–18.4)	77.6±4.57 (67.5–85)	13.9±1.63 (10.2–16.3)	46.7±5.67 (34–58)	61.5±2.2 (56.1–64.8)	3.9±0.43 (3.1–5.1)	140±6.5 (125–155)
RsBs	20.5±2.15 (17.4–25.9)	19.4±0.25 (18.4–19.9)	74.2±3.42 (65–80)	12.7±1.66 (8.2–15.3)	46.5±4.36 (37–58)	63.3±2.56 (59.2–67.3)	4.3±0.53 (3.1–5.1)	145.2±9.18 (125.5–165.2)
RsG	23.8±2.4 (19.1–27.5)	18.3±0.52 (17.3–19.4)	81.3±4.39 (72.5–90)	14.2±1.45 (11.2–16.3)	47.1±3.46 (40–53)	63.2±1.91 (60.2–67.3)	4.3±0.58 (3.1–5.1)	145.9±5.04 (135.7–157.1)
RsGs	19.7±1.74 (16.5–26.2)	19.1±0.42 (18.4–19.4)	72.3±3.64 (63.8–78.8)	13.1±1.06 (10.7–15.3)	46.4±3.19 (41–53)	62.7±2.32 (58.1–67.3)	4.4±0.53 (3.1–5.1)	140±5.83 (129.5–151)
RsD	24.6±2.49 (21.2–30.4)	19.1±0.6 (18.4–20.4)	76.5±4.01 (67.5–81.3)	12.7±1.03 (11.2–15.3)	46.4±4.43 (39–58)	63.2±1.86 (61.2–67.3)	4.3±0.45 (4.1–5.1)	133.9±5.44 (125–143.8)
RsDs	19.8±1.08 (17.8–22.6)	19.3±0.42 (18.4–20.4)	68.8±3.39 (59.5–76.3)	11.8±1.16 (8.7–14.3)	45.5±3.43 (35–51)	61.1±2.57 (54.1–66.3)	4.6±0.5 (4.1–5.1)	130.2±6.89 (121.4–152)
RsE	21.3±2.04 (16.8–23.6)	18.6±0.45 (18.4–19.4)	75.9±3.93 (70–85)	13.3±1.22 (11.2–16.3)	47.3±4.36 (40–60)	60.8±1.94 (58.1–64.3)	3.7±0.5 (3.1–4.1)	137±6.35 (126.5–149.9)
RsEs	20.5±2.49 (16.9–26.4)	18.9±0.51 (18.4–19.9)	71.9±5.26 (61.3–80)	12.9±1.49 (10.2–16.8)	45.5±4.16 (35–52)	62.7±2.49 (57.1–67.3)	4.3±0.41 (4.1–5.1)	142.2±7.27 (126.5–156.1)
RsH	24.7±2.75 (19.5–28.9)	19±0.5 (18.4–19.4)	86.9±4.4 (80–95)	10.3±1.62 (6.6–13.3)	46.1±4.17 (38–53)	64±2.52 (57.1–68.3)	4.3±0.42 (4.1–5.1)	137.4±6.97 (126.5–152)
RsHs	18.4±1.49 (15.7–23.1)	19.1±0.5 (17.3–19.4)	81.6±4.17 (73.8–88.8)	8.9±1.61 (5.6–12)	48.1±3.61 (41–54)	62.7±2.41 (59.2–70.4)	4.6±0.49 (4.1–5.1)	139±8.68 (124.4–159.1)
RsI	20.9±2.23 (17.7–25.6)	18.5±0.6 (17.3–19.4)	71.2±3.81 (62.5–77.5)	13.8±1.41 (11.2–17.3)	43.5±3.56 (38–49)	57.6±1.78 (54.1–60.2)	4.1±0.62 (3.1–5.1)	132.5±5.92 (119.3–145.9)
RsIs	20.2±2.06 (17.5–25.2)	18.9±0.55 (17.3–19.4)	70.7±4.5 (55–76.3)	12.3±1.6 (8.2–14.8)	44.2±4.62 (33–54)	61±2.25 (53–64.3)	4.3±0.39 (4.1–5.1)	135.2±6.78 (119.3–148.9)
RsJ	27.5±2.92 (22.6–32.7)	18.1±0.69 (17.3–19.4)	83.5±4.23 (72.5–90)	12.6±1.75 (8.7–15.3)	41.8±2.59 (37–45)	62±1.79 (59.2–65.3)	4.5±0.55 (3.6–5.1)	130±4.37 (122.4–138.2)
RsJs	19.8±1.81 (17.5–24.6)	18.5±0.42 (17.3–19.4)	81.6±3.84 (75–88.8)	10±1.94 (6.1–14.3)	44.7±4.17 (39–55)	59.2±1.87 (56.1–62.2)	4.3±0.51 (3.1–5.1)	127.6±6.6 (118.3–143.8)
RsV	25.2±2.82 (21.8–31.7)	18.8±0.75 (17.3–19.4)	79.1±4.93 (67.5–87.5)	11.6±1.13 (9.2–13.3)	48.5±4.62 (38–57)	62.3±1.32 (59.2–65.3)	4.3±0.34 (4.1–5.1)	147±8.66 (132.6–164.2)
RsVs	20.8±1.92 (18–26)	19.2±0.35 (18.4–19.4)	75.9±5.56 (67.5–86.3)	11.4±1.66 (8.2–14.3)	46.3±4.78 (38–56)	62.8±1.89 (58.1–67.3)	4.4±0.42 (4.1–5.1)	141.3±5.86 (129.5–153)
RsW	29.4±3.24 (25–36.2)	18.4±0.47 (17.3–19.4)	86.9±4.62 (77.5–95)	11±1.73 (8.2–15.3)	41.4±4.45 (36–53)	61.7±2.05 (55.1–64.3)	4.4±0.48 (4.1–5.1)	128.1±7.37 (114.2–151)
RsWs	22.5±3.49 (17.9–30.4)	18.3±0.41 (17.3–19.4)	80.2±4.57 (71.3–86.3)	10.1±1.96 (6.6–14.3)	42.8±3.92 (36–52)	59.2±1.92 (55.1–62.2)	4.1±0.43 (3.1–5.1)	127.8±9.39 (113.2–146.9)
RsY	27.8±3.68 (21.9–34.5)	19.6±0.43 (19.4–20.4)	77±4.22 (72.5–85)	12.7±1.31 (10.7–14.8)	46.4±2.59 (42–51)	64.2±1.76 (61.2–68.3)	4.6±0.51 (4.1–5.1)	136.1±6.67 (128.5–146.9)
RsYs	19.9±1.21 (17.7–23.5)	19.4±0.46 (18.4–20.4)	74.9±3.27 (69.5–81.3)	12.4±1.32 (10.2–15.8)	47.9±2.98 (41–53)	63.7±1.96 (59.2–67.3)	4.6±0.7 (3.1–6.1)	140.3±5.44 (125.5–153)
RsL	20.3±1.47 (18–23.2)	19.3±0.48 (18.4–20.4)	72.9±5.59 (65.5–90)	12.3±0.91 (10.7–13.8)	49±4.9 (38–58)	63.8±2.58 (58.1–67.3)	4.5±0.51 (4.1–5.1)	147±9.47 (120.4–161.2)
RsLs	20.6±2.22 (18.3–30)	19.2±0.6 (18.4–20.4)	72.1±4.4 (64.8–83.8)	12±1.22 (9.2–14.3)	48.6±5.13 (38–57)	59.5±2.02 (56.1–64.3)	4±0.31 (3.1–5.1)	133.8±10.06 (106.1–156.1)
RsM	18.4±1.1 (15.7–20.4)	18.6±0.53 (17.3–19.4)	71.6±3.18 (66.3–77.5)	12.1±1.25 (9.2–14.3)	45.3±3.39 (39–52)	59.1±2.14 (55.1–63.2)	3.9±0.63 (3.1–5.1)	133.3±7.06 (123.4–144.8)
RsMs	19.9±1.15 (17.7–22.6)	19.3±0.6 (18.4–21.4)	71.7±4.43 (65–83.8)	12.3±1.22 (10.2–14.8)	46.2±3.53 (40–55)	62.1±3.42 (56.1–70.4)	4.6±0.47 (4.1–5.1)	137.4±8.07 (122.4–155)
RsN	21.3±1.99 (19–27.6)	18.9±0.62 (18.4–20.4)	76.9±3.96 (70–82.5)	13.5±1.49 (10.2–16.3)	44.4±3.15 (39–51)	60.8±2.33 (56.1–64.3)	4.1±0.23 (4.1–5.1)	139.2±6.68 (128.5–149.4)
RsNs	19.5±1.86 (16–24.3)	19.2±0.39 (18.4–19.4)	73.5±3.1 (68.8–81.3)	13.2±1.17 (10.7–15.3)	47.8±4.97 (37–56)	63±2.81 (58.1–72.4)	4.3±0.45 (3.1–5.1)	142.8±7.96 (128.5–168.3)
RsXJ	28.1±3.03 (22.8–34)	17.7±0.51 (17.3–18.4)	84.8±2.8 (80–90)	8±2.96 (5.6–19.4)	37.5±1.61 (35–40)	59.9±3.07 (53–65.3)	3.7±0.5 (3.1–4.1)	130.8±3.65 (122.4–136.7)
RsXjs	20.1±1.42 (16.9–23.6)	18.4±0.38 (17.3–19.4)	77.5±3.9 (67.5–86.3)	7.5±1.44 (3.6–10.2)	37.9±3.61 (24–46)	61±2.08 (58.1–65.3)	4.1±0.4 (3.1–5.1)	127.1±6.67 (116.3–144.8)

^a^ Code of progeny from 30 females (Codes as RsA et al) and a single young female nematode (Coded as RsAs et al) of different population of *Radopholus similis*, RsA/RsAs: intercepted from *Calathea zebrine* in Netherlands; RsB/RsBs: collected from *Ravenea rivularis* in China; RsC/RsCs: intercepted from *Calathea* sp. in Netherlands; RsD/RsDs, RsG/RsGs: collected from *Chamaedorea cataractarum* in China; RsE/RsEs, RsH/RsHs: collected from *Philodendron* sp.in China; RsI/RsIs, RsJ/RsJs, RsV/RsVs, RsW/RsWs, RsY/RsYs: collected from *Anthurium andraeanum* in China; RsK/RsKs, RsP/RsPs: collected from *Calathea zebrine* in China; RsL/RsLs: collected from *Epipremnum aureum* in China; RsM/RsMs:intercepted from *Stranvaesia* sp. in Malaysia; RsN/RsNs: collected from *Chrysalidocarpus lutescens* in China; RsT/RsTs: collected from *Calathea* sp. in China; RsS/RsSs: collected from *Calathea makoyana* in China; RsXj/RsXjs: intercepted from *Zingiber officinale* Roscoe in Singapore.

**Table 3.** Morphometrics of *Radopholus similis* males from offspring of mixed females and single female from 20 populations (n = 20), respectively (measurements: µm).

Code^b^	*L*	*a*	*c*	*c*’	*stylet*	*MB*	genital length	testis length
RsA	660.2±20.45 (601.2–692.5)	37.2±2.67 (32.1–41)	7.5±0.36 (6.8–8.3)	6.8±0.49 (6–7.7)	13.7±0.9 (11.2–15.3)	52.9±2.27 (49–57.1)	186.6±16.11 (162.5–222.5)	54.2±10.23 (33.7–79.6)
RsAs	708.5±21.67 (660–763.8)	43±3.11 (37.1–47.6)	7.4±0.93 (4.1–8.1)	7.5±1.47 (6.3–13.1)	13.3±0.83 (11.2–14.3)	58.9±2.44 (53–64.3)	179.1±16.41 (146.9–217.5)	51.2±10.18 (30.6–78.5)
RsK	644.1±40.47 (577.5–770)	38.1±2.49 (31.8–44)	7.3±0.32 (6.3–7.7)	6.9±0.49 (6.2–7.8)	12±0.73 (11.2–13.3)	53.9±2.03 (52–60.2)	206.3±18.45 (180–245)	59.5±17.08 (27.5–89.8)
RsKs	618.8±33.06 (542.5–732.5)	38±2.85 (32.5–43.1)	7.4±0.45 (6.4–8.7)	6.5±0.51 (5.7–7.7)	11.9±0.94 (10.2–13.3)	54.8±2.29 (50–59.2)	191.5±23.06 (127.5–232.6)	46.3±18.76 (24.5–108.1)
RsP	647.9±22.15 (612.5–702.5)	37.3±1.8 (32.9–40.8)	8.4±0.35 (7.9–9.4)	5.7±0.29 (5–6.1)	11.6±0.57 (11.2–13.3)	56±1.79 (52–58.1)	206.4±23.65 (155–240)	77.1±14.19 (54.1–98.9)
RsPs	632.6±24.35 (567.5–676.3)	38.6±2.41 (32.9–43.2)	8.5±0.37 (7.8–9.3)	5.7±0.35 (5.2–6.3)	11.9±1.24 (10.2–14.3)	59.4±2.50 (54.1–63.2)	196.4±23.71 (157.5–247.5)	57.4±12.82 (27.5–90.8)
RsC	666.4±26.44 (605–707.5)	41.4±2.8 (38.4–47.5)	7.7±0.23 (7.4–8.2)	6.8±0.34 (6.4–7.5)	13.2±0.34 (12.2–13.8)	55.9±3.35 (51–65.3)	218.8±27.7 (163.2–250.9)	63.6±12.7 (41.8–84.7)
RsCs	662.8±30.59 (613.8–730)	40.9±2.41 (36.2–45.8)	7.7±0.3 (7.2–8.5)	6.8±0.42 (6–7.7)	13.5±0.48 (12.8–14.8)	57.6±3.07 (52–62.2)	174.2±26.64 (122.4–232.5)	38.2±8.39 (22.4–56.1)
RsS	656.3±16.05 (621.3–677.5)	38.1±1.61 (35.5–41)	7.6±0.26 (7.28.3-)	6.6±0.41 (5.8–7.4)	13.6±0.74 (12.2–14.8)	55.6±2.61 (53–63.2)	215.2±14.67 (192.8–247.9)	57.3±6.66 (45.9–69.4)
RsSs	670.3±26.22 (622.5–717.5)	38.5±3.27 (33.1–43.8)	7.6±0.43 (6.6–8.7)	6.8±0.65 (5.2–8.1)	13.2±1.36 (10.2–14.8)	58.4±3.28 (50–62.2)	208.4±19.83 (170–257.5)	49.6±17.73 (27.5–111.7)
RsT	615.3±29.52 (548.8–680)	37.4±2.38 (33.4–42)	8.2±0.27 (7.7–8.6)	5.8±0.26 (5.3–6.2)	12.6±1.32 (11.2–15.3)	55.5±2.32 (50–60.2)	180.8±23.16 (142.5–225)	49.6±17.65 (23.5–96.9)
RsTs	615.2±29.1 (545–695)	37.3±2.84 (31.1–43)	8.4±0.4 (7.8–9.3)	5.8±0.28 (5.4–6.3)	11.9±2.19 (9.2–16.3)	60.7±2.77 (55.1–65.3)	190.5±19.95 (142.8–230)	52.3±19.84 (12.2–107.1)
RsB	607.1±15.75 (571.3–637.5)	32.5±1.93 (29.1–36.8)	8±0.33 (7.4–8.6)	5.9±0.42 (5.2–6.8)	12.6±1.46 (10.2–16.3)	54.7±2.08 (51–59.2)	174.1±21.71 (132.5–217.5)	39.1±19.73 (16.3–78.5)
RsBs	613.7±20.8 (555–662.5)	36.4±2.8 (31.1–42.1)	8.2±0.31 (7.7–8.9)	5.8±0.39 (5–6.6)	14.1±0.7 (12.2–15.3)	57.7±2.49 (53–62.2)	201.7±18.7 (167.5–230)	75±25.33 (31.6–123.4)
RsG	621.5±21.13 (572.5–657.5)	35.1±2.2 (30.8–38.6)	8.2±0.33 (7.7–8.8)	5.8±0.43 (5.2–6.6)	14.1±0.53 (13.3–15.3)	56.3±2.72 (51–62.2)	197.5±13.45 (180–222.5)	57.1±13.56 (35.7–84.7)
RsGs	605.9±20.04 (550–643.8)	37±2.59 (33.7–42.9)	8.4±0.31 (7.8–9.2)	5.7±0.38 (4.1–6.2)	11.9±1.56 (10.2–14.3)	56.8±2.81 (51–62.2)	170.2±17.31 (133.6–215)	42±14.63 (21.4–95.9)
RsD	620.3±26.7 (575–670)	34.4±2.7 (31.3–42.3)	8.4±0.34 (7.6–8.9)	5.5±0.37 (5.1–6.2)	12.6±0.94 (11.2–14.3)	55.7±1.8 (52–59.2)	190.8±16.55 (170–220)	68.7±10.39 (34.7–82.6)
RsDs	594.8±23.14 (547.5–632.5)	38±1.84 (33.1–42)	8.6±0.49 (8–9.9)	5.5±0.37 (4.8–6.2)	12.4±1.68 (10.2–16.3)	58.1±2.53 (53–62.2)	179.7±22.53 (137.5–224.4)	50.3±16.14 (27.5–105.1)
RsE	611.4±21.39 (556.3–641.3)	34.1±1.4 (30.5–36.5)	8±0.24 (7.7–8.7)	5.8±0.37 (5.3–6.4)	12.5±0.71 (11.2–13.3)	54.1±2.21 (9–58.1)	190.1±12.02 (160–205)	59.5±12.47 (27.5–83.6)
RsEs	616.8±33.67 (540.5–723.8)	38.4±2.5 (32.6–44.5)	8.4±0.47 (7.6–10.3)	5.8±0.3 (5.1–6.3)	13.5±1.54 (10.2–15.3)	58.3±2.18 (53–63.2)	175.5±22.26 (125–217.5)	46±14.18 (23.5–84.7)
RsH	676.9±26.49 (631.3–717.5)	35.4±2.1 (31.9–38.7)	7.4±0.24 (6.7–7.7)	6.9±0.51 (6.2–8.1)	13±0.87 (11.2–14.3)	55.1±1.4 (52.5–57.1)	195.4±16 (167.5–227.5)	49.7±9.07 (35.7–71.4)
RsHs	651.6±41.32 (527.5–710)	39±3.08 (33.3–47.3)	7.6±0.27 (6.9–8.3)	7±0.5 (6.2–8)	12±1.22 (9.2–14.3)	58.3±2.7 (50–65.3)	188.3±16.89 (155–232.6)	45.3±20.32 (23.5–95.9)
RsI	612±20.85 (572.5–641.3)	38.6±1.5 (35.4–42.3)	8.3±0.29 (7.7–8.8)	6.1±0.34 (5.4–6.7)	12.9±0.67 (11.2–13.8)	53±1.87 (50–56.1)	175.3±21.38 (140–215)	51.9±12.75 (30.6–77.5)
RsIs	609±22.36 (551.3–641.3)	37.5±2.19 (33.9–41.8)	8.4±0.29 (7.8–9.2)	5.8±0.3 (5.2–6.4)	12.4±1.22 (10.2–14.3)	56.5±3.04 (49–61.2)	173.3±18.33 (122.5–207.1)	44.3±12.74 (20.4–84.7)
RsJ	615.5±33.84 (536.3–672.5)	35.9±2.1 (30.6–40.7)	7.4±0.26 (6.9–7.9)	6.5±0.47 (5.6–7.3)	12.2±0.89 (11.2–14.3)	52.5±2.2 (46.9–58.1)	202.5±21.52 (162.5–260)	61.8±10.88 (27.5–78.5)
RsJs	605.9±29.72 (554.5–650)	36.9±2.78 (31.7–42.8)	7.5±0.27 (6.8–8)	6.2±0.42 (5.4–7.1)	12.4±0.94 (11.2–15.3)	55.1±2.16 (52–60.2)	193.7±19.45 (160–233.6)	41.6±13.32 (27.5–82.6)
RsV	614.8±22.17 (570–650)	36.1±2.3 (31.9–41.6)	8.3±0.26 (7.8–8.8)	5.7±0.47 (5.1–7.4)	14.8±0.69 (13.3–15.3)	56.6±2.74 (52–62.2)	196.7±16.22 (161.2–222.4)	74.4±16.95 (37.7–98.9)
RsVs	620.8±28.16 (568–713.8)	38±1.83 (35.4–41)	8.3±0.34 (7.5–9)	5.8±0.47 (5.1–7.1)	11.6±1.9 (9.2–14.3)	58.5±1.63 (55.1–63.2)	188±15.62 (150–217.5)	52.7±14.08 (16.3–82.6)
RsW	602.2±20.24 (567.5–627.5)	37.1±3.7 (31.4–41.7)	7.3±7.28 (6.5–7.8)	6.5±6.57 (5.5–7.4)	11.1±0.71 (10.2–12.2)	53.1±53.24 (51–57.1)	196.1±18.2 (157.5–232.5)	47.5±8.7 (32.6–62.2)
RsWs	600.2±30.42 (555–675)	35.1±2.33 (30.1–39.1)	7.5±0.3 (6.9–8.1)	6.3±0.39 (5.3–7.3)	13.4±0.85 (12.2–15.3)	54.7±2.17 (50–58.1)	194.2±13.23 (176.5–227.5)	45.8±21.71 (22.4–119.3)
RsY	619.5±18.67 (578.8–648.8)	35.5±2.0 (31.5–40.4)	8.4±0.28 (8–9)	5.5±0.5 (4.5–6.6)	14.8±0.68 (13.8–16.3)	55.5±1.96 (52–58.1)	194.7±15.86 (155–222.4)	65.8±17.7 (34.7–87.7)
RsYs	632±26.48 (586.3–687.5)	37.7±2.58 (29.3–41.7)	8.5±0.3 (8.1–9.2)	5.8±0.7 (4.9–9.1)	12±1.78 (10.2–15.3)	60.3±2.04 (57.1–64.3)	202.4±21.84 (147.5–253)	59.9±16.67 (30.6–104)
RsL	618.3±22.94 (581.3–658.8)	38.8±2.7 (34.6–43.6)	8.3±0.36 (7.7–9)	5.6±0.31 (5.1–6.2)	15.1±0.83 (13.3–16.3)	61.3±1.8 (58.1–64.3)	182.8±16.68 (143.8–227.5)	38.1±9.71 (24.5–56.1)
RsLs	611.9±30.33 (560–658.8)	36.8±1.98 (33.5–39.4)	8.2±0.34 (7.7–8.7)	5.6±0.33 (5.3–6.3)	11.3±1.41 (9.7–14.3)	57.2±2.85 (47.9–61.2)	201.6±28 (152.5–247.5)	66.7±20.69 (31.6–106.1)
RsM	638.5±16.48 (610–662.5)	36.2±2.1 (32.2–40.7)	8.5±0.27 (7.9–9)	5.7±0.34 (5.3–6.5)	13.1±1.03 (11.7–15.3)	55.1±2.17 (52–60.7)	195.2±21.54 (157.5–237.5)	64.5±17.27 (41.8–104)
RsMs	622±27.21 (577.5–687.5)	37.6±1.97 (33.7–40.3)	8.4±0.34 (7.7–9.2)	5.7±0.35 (4.9–6.5)	13.9±1.08 (11.2–15.3)	59.1±2.63 (54.1–65.3)	188.8±21.89 (147.5–227.5)	59.5±18.89 (17.3–93.8)
RsN	619.4±22.21 (572.5–655)	37.6±2.1 (33.1–41.2)	8.1±0.37 (7.5–9)	6±0.32 (5.6–6.8)	13.3±0.92 (11.2–14.3)	55.4±2.18 (51–60.2)	189.4±22.92 (147.5–235)	57.4±14.3 (28.6–80.6)
RsNs	605.4±18.97 (567.5–637.5)	38.6±2.62 (32.5–43.8)	8.2±0.31 (7.5–9.2)	5.9±0.43 (5.1–7.3)	13±2.18 (9.2–15.3)	58.8±3.37 (53–66.3)	185.9±28.28 (150–244.8)	60.1±28.12 (28.6–124.4)
RsXJ	617.2±29.31 (570–712.5)	32.8±3.3 (30.3–37.8)	7.7±7.8 (7.2–8.6)	6±6.09 (5.5–6.7)	11.6±0.49 (11.2–12.2)	52.7±53.22 (51–61.2)	187±14.09 (160–205)	51.2±11.37 (35.7–70.4)
RsXjs	624.3±15.12 (583.8–645)	36.1±1.81 (31.3–39.8)	7.7±0.22 (7.2–8.2)	6±0.39 (4.9–6.8)	12.2±1.88 (10.2–15.3)	56.3±2.18 (51–60.2)	203.2±9.63 (177.5–222.5)	57.7±10.23 (41.8–75.5)

Code	*Tail*	*h*	gubernaculum length	spicule length	distance from anterior to excretory core	lip heigt	body diameter	number of genital papilla
RsA	88.8±5.84 (77.5–98.8)	7.2±1.07 (5.1–9.2)	10.7±0.68 (9.2–11.7)	18.7±1.09 (16.3–20.4)	89.3±2.86 (84.7–94.9)	6.3±0.24 (6.1–6.6)	17.8±1.32 (16.3–20)	0–6
RsAs	97.6±18.2 (85–163.8)	7.5±1.43 (5.6–12.2)	11.1±0.9 (8.2–12.4)	19.8±1.7 (14.3–21.4)	96.1±3.33 (87.7–104)	6.2±0.25 (5.6–6.6)	16.5±1.22 (15–18.8)	0–6
RsK	88.8±5.35 (77.5–101.3)	6.2±1.09 (4.6–8.7)	10.7±0.6 (9.7–12)	18.4±1.77 (16.3–23.5)	92.2±4.03 (86.2–102)	6±0.37 (5.6–7.1)	16.9±1.08 (15–18.8)	0
RsKs	83.3±5.62 (72.5–96.3)	6.5±1.38 (4.6–9.7)	10.1±0.66 (8.2–11.2)	18.1±1.23 (16.3–21.4)	88.8±3.21 (81.6–94.9)	5.9±0.3 (5.3–6.6)	16.4±1.43 (14.5–20)	0–1
RsP	77±3.48 (72.5–87.5)	7±1.02 (5.1–8.7)	11.1±0.48 (10.2–12.2)	19.7±1.45 (15.3–21.4)	92.4±3.73 (83.6–97.9)	6.1±0.11 (5.6–6.1)	17.4±1.09 (15–20)	2–9
RsPs	74.8±3.84 (66.3–82.5)	6.5±1.25 (4.1–9.2)	11.2±0.52 (9.7–12.8)	20±0.93 (18.4–21.4)	91.1±3.58 (85.7–99.5)	6.1±0.25 (5.1–6.6)	16.4±1.27 (15–19.8)	8
RsC	86.8±4.55 (77.5–95)	8.5±2.47 (4.6–12.8)	10.8±0.56 (9.7–11.7)	18.9±0.9 (17.3–20.4)	91±4.07 (84.2–97.9)	5.9±0.34 (5.1–6.2)	16.2±1.43 (13.8–17.8)	0–3
RsCs	86±4.64 (78.8–96.3)	7.2±1.59 (5.1–13.3)	10.8±0.68 (9.7–11.7)	18.9±1.2 (16.3–22.4)	91.7±4.28 (84.7–101)	6±0.25 (5.6–6.6)	16.2±1.01 (14.8–18.8)	0–3
RsS	86±3.79 (80–92.5)	7.7±1.6 (5.1–10.7)	10.8±0.62 (9.7–11.7)	18.5±0.6 (17.9–19.4)	93.1±3.95 (86.2–100)	6±0.21 (5.6–6.1)	17.3±0.47 (16.3–17.8)	0
RsSs	83.9±5.79 (72.5–93.8)	6.6±1.61 (3.6–9.7)	11.2±0.9 (9.2–12.2)	19.3±1.14 (16.3–21.9)	94.7±3.39 (86.7–100)	6.1±0.19 (5.6–6.6)	17.5±1.27 (15–20)	0
RsT	75.4±3.12 (70–83.8)	7±1.05 (5.1–9.2)	10.8±1.06 (8.2–12.2)	20.1±1.43 (17.3–23)	95.9±5.39 (87.7–109.1)	5.9±0.33 (5.3–6.6)	16.5±1.18 (15–18)	0–5
RsTs	73.1±3.22 (67.5–78.8)	7.6±1.06 (5.6–9.7)	10.6±0.72 (8.7–11.7)	20.1±1.28 (16.3–22.4)	94.7±3.8 (87.7–102.5)	5.9±0.25 (5.6–6.1)	16.6±1.38 (15–20)	4–5
RsB	76.3±3.33 (67.5–81.3)	7.8±1.24 (5.6–10.2)	10.8±1.05 (9.2–12)	20.5±1.55 (17.3–23.5)	90.4±3.11 (83.6–96.4)	5.9±0.26 (5.6–6.1)	18.8±1.2 (16.3–20.5)	4–6
RsBs	74.8±3.73 (66.3–82.5)	7.6±1.38 (5.1–10.7)	11±0.63 (8.7–12)	20.2±1.02 (18.4–22.4)	95.1±3.25 (89.8–102)	5.8±0.31 (5.1–6.1)	17±1.54 (15–20)	4–5
RsG	76.1±3.73 (67.5–82.5)	7.9±0.82 (6.6–9.2)	11.1±0.63 (10.2–12.2)	20.5±1.5 (18.4–23.5)	94.2±2.58 (89.8–98.9)	6±0.24 (5.6–6.1)	17.7±0.98 (16.3–20)	8
RsGs	72.1±3.02 (67.5–78.8)	7.4±0.92 (5.1–9.2)	10.8±0.72 (8.7–11.7)	20.1±1.42 (17.3–25.5)	91.2±4.53 (78.5–97.9)	5.7±0.29 (5.1–6.1)	16.4±1.1 (14.8–18)	8
RsD	74.2±3.9 (66.3–83.8)	7.9±1.05 (4.6–9.2)	11.4±0.58 (10.2–12.2)	21±1.41 (18.4–24.5)	92.7±4.26 (85.2–100)	6.1±0.3 (5.2–6.5)	18.1±1.56 (15–20)	0–6
RsDs	69.4±4.28 (60–76.3)	7±0.83 (5.6–8.7)	10.9±0.6 (9.4–12.2)	19.8±0.97 (17.3–21.4)	90.9±3.33 (84.7–96.4)	5.9±0.3 (5.1–6.1)	15.7±0.82 (14.5–18)	2–7
RsE	76.4±3.11 (71.3–82.5)	7.7±0.6 (6.1–8.7)	11±0.48 (10.2–11.7)	19.8±1.09 (18.4–22.4)	91.3±4 (84.2–98.4)	5.9±0.25 (5.6–6.1)	17.9±0.85 (16.3–20)	4–5
RsEs	73.9±3.83 (65–81.3)	7.3±0.94 (5.1–9.2)	10.7±0.7 (9.2–11.7)	19.8±1.07 (17.3–21.4)	94.6±4.13 (85.7–103)	5.9±0.29 (5.1–6.1)	16.1±1.27 (13.8–19.5)	0–6
RsH	91.1±4.07 (82.5–98.8)	6.8±1.92 (4.1–10.7)	11.2±1.03 (9.2–12.2)	19.2±1.26 (17.3–22.4)	94.5±5.05 (83.1–106.1)	6±0.21 (5.6–6.1)	19.2±0.92 (17.5–21.3)	5
RsHs	86.3±5.99 (71.3–96.3)	5.1±1.03 (3.1–7.7)	10.4±0.81 (8.7–11.7)	18.7±1.49 (16.3–23.5)	91.7±5.11 (78–102)	5.7±0.29 (5.1–6.1)	16.8±1.36 (15–18.8)	0–4
RsI	74.2±2.91 (67.5–78.8)	7.6±0.95 (6.1–9.2)	10.9±0.65 (9.7–11.7)	19.7±0.99 (18.4–21.4)	91.1±3.62 (84.7–98.9)	5.6±0.35 (5.1–6.1)	15.9±0.68 (15–17)	5
RsIs	72.7±4.01 (62.5–79.8)	7.8±1.42 (6.1–12.8)	11.1±0.5 (9.4–12.2)	20.4±0.92 (18.4–22.4)	89.5±3.9 (82.1–97.9)	5.9±0.25 (5.6–6.1)	16.3±0.91 (15–17.5)	3–5
RsJ	82.9±3.42 (77.5–91.3)	7.5±1.85 (4.6–12.2)	10.1±0.66 (9.2–11.2)	17.2±0.78 (16.3–19.4)	87.9±4.06 (83.6–96.9)	6.1±0.35 (5.1–7.1)	17.2±1.08 (15–18.8)	0
RsJs	80.5±4.63 (73.8–87.5)	6.9±1.39 (4.6–10.2)	10.1±0.58 (9.2–11.2)	18.4±0.93 (17.3–20.4)	88.6±4.06 (80.6–95.9)	6.1±0.17 (5.6–6.2)	16.5±1.27 (13.8–18.8)	0
RsV	74±2.62 (68.8–80)	6.4±1.03 (4.6–8.2)	11±0.47 (10.2–11.7)	20.1±0.87 (18.4–21.9)	93.3±3.47 (85.7–98.9)	5.9±0.26 (5.6–6.1)	17.1±1.28 (15–20)	4–7
RsVs	74.6±5.12 (66.3–88.8)	6.6±1.31 (3.6–9.7)	11±0.58 (9.2–11.7)	20.3±1.05 (17.3–21.4)	93.2±3.79 (85.7–103)	5.8±0.33 (5.1–6.6)	16.4±1.04 (15–17.5)	4
RsW	83.1±4.54 (75–92.5)	7±1.1 (5.1–9.2)	10.2±0.88 (8.2–11.2)	17.4±1.37 (15.3–20.4)	87.6±2.52 (82.6–93.3)	6±0.19 (5.6–6.1)	16.3±1.11 (15–18.8)	0
RsWs	80.3±4.56 (71.3–92.5)	6.5±1.76 (3.6–10.1)	10±0.68 (8.7–11.2)	18.1±1.08 (16.3–20.4)	86.3±4.92 (74.5–95.9)	6±0.31 (5.1–6.3)	17.1±1.02 (15–18.8)	1
RsY	73.7±3.16 (67.5–78.8)	6.8±1.28 (4.6–9.7)	11.5±0.4 (10.7–12.1)	20.8±1.1 (18.4–22.4)	96.9±3.96 (88.2–104.6)	6.1±0.05 (6.1–6.3)	17.5±1.08 (15.3–20)	0
RsYs	74.6±3.96 (66.3–81.3)	7.5±1.12 (5.6–10.2)	11±0.69 (8.7–11.7)	20.5±1.03 (16.3–22.4)	95.4±2.47 (89.8–100)	6±0.3 (5.1–6.6)	16.9±1.24 (15–20)	0
RsL	74.8±4.24 (67.5–83.8)	6.8±1.25 (4.6–10.2)	10.9±0.51 (10.2–12)	20±0.71 (18.9–21.9)	94.1±2.94 (87.7–97.9)	5.9±0.27 (5.3–6.1)	16±1.16 (15–18)	2–6
RsLs	73.5±4.22 (67–81.3)	6.9±1.4 (4.6–11.7)	11.2±0.53 (9.7–12)	20.5±1.01 (18.4–22.4)	89.9±3.27 (82.6–94.9)	6±0.24 (5.6–6.1)	17.4±1.42 (14.8–20)	6
RsM	75.3±2.94 (67.5–81.3)	7.1±1.03 (5.6–9.2)	11.4±0.53 (10.2–12.2)	20.2±1.12 (18.4–22.4)	95.8±3.53 (90.8–104)	6±0.23 (5.6–6.1)	17.7±1.25 (15–20.5)	7–8
RsMs	74.5±4.89 (67.5–86.3)	6.6±1.22 (4.6–9.2)	10.4±1.1 (7.7–12)	20.2±2.01 (15.3–24.5)	94.1±3.84 (87.7–102)	6±0.27 (5.6–6.6)	16.6±1.06 (15–18.8)	6–8
RsN	76.7±4.27 (70–86.3)	6.9±1.25 (4.6–9.2)	10.9±0.61 (9.7–12.2)	20±1.15 (18–24)	92.4±4.03 (85.7–100)	5.9±0.26 (5.6–6.1)	16.5±0.75 (15–18)	4–6
RsNs	73.5±3.43 (66.3–83.8)	7.2±0.8 (6.1–9.2)	10.7±0.76 (8.2–11.7)	20±1.1 (17.3–21.4)	94.1±3.94 (88.7–102)	5.8±0.31 (5.1–6.1)	15.7±1.18 (13.8–18.8)	3–6
RsXJ	79.8±3.55 (72.5–86.3)	6±1.26 (4.6–9.7)	10.6±0.44 (9.7–11.2)	19.4±1.4 (17.3–19.8)	90.3±3.23 (84.7–95.9)	6±0.29 (5.1–6.1)	18.8±1.09 (16.3–20)	0
RsXjs	80.8±2.87 (73.8–85.5)	6±1.06 (4.6–8.2)	10.4±0.73 (8.2–11.7)	19.3±1.11 (15.3–20.9)	92.9±2.6 (88.7–97.4)	5.8±0.3 (5.3–6.1)	17.3±0.89 (15–20)	0

^b^ Code of progeny from 30 females (Codes as RsA et al) and a single young female nematode (Coded as RsAs et al) of different population of *Radopholus similis*, RsA/RsAs: intercepted from *Calathea zebrine* in Netherlands; RsB/RsBs: collected from *Ravenea rivularis* in China; RsC/RsCs: intercepted from *Calathea* sp. in Netherlands; RsD/RsDs, RsG/RsGs: collected from *Chamaedorea cataractarum* in China; RsE/RsEs, RsH/RsHs: collected from *Philodendron* sp. in China; RsI/RsIs, RsJ/RsJs, RsV/RsVs, RsW/RsWs, RsY/RsYs: collected from *Anthurium andraeanum* in China; RsK/RsKs, RsP/RsPs: collected from *Calathea zebrine* in China; RsL/RsLs: collected from *Epipremnum aureum* in China; RsM/RsMs: intercepted from *Stranvaesia* sp. in Malaysia; RsN/RsNs: collected from *Chrysalidocarpus lutescens* in China; RsT/RsTs: collected from *Calathea* sp. in China; RsS/RsSs: collected from *Calathea makoyana* in China; RsXj/RsXjs: intercepted from *Zingiber officinale* Roscoe in Singapore.

### Morphological observations of progeny of 30 females

Females. The shortest individual female (513.8 µm) was found in the RsK population from *Calathea zebrina*, and the shortest females with average length of 656.7 µm were from the RsM population from *Stranvaesia* sp.. The longest individual female (843.8 µm) and the longest females with average length of 795.9 µm were found in the RsC population from *Calathea* sp.. Head diameter and height were almost identical in all the populations and ranged from 9.8×4.3 µm to 9.1×4 µm. The number of head annuli varied in and among populations, with 2 annuli in the RsS and RxXj populations, 3–4 annuli in the RsL, RsT, RsV and RsY populations, and 3 annuli in the remaining populations. The stylet length varied from 17.3 μm to 19.6 μm. Tail length and shape varied in and among populations. The longest tails with average length of 90.9 μm was found in the RsC population. The shortest tails with average length of 71.2 μm was found in the RsI population. The most and least tail annulations (61 and 32, respectively) were all found in the RsP population. The average length of the hyaline part of the tail of all populations was longer than 5.6 μm, 97.5% of these individuals was longer than 7 μm, and only 0.5% was 5.6 μm. In addition, the shortest and longest hyaline part of tail were all from the RsH population (3.1 μm and 10.7 μm, respectively). Tail shape showed four differenttypes (I–IV). The type I tail is conoid, slightly or abruptly slender to tail terminus, tail terminus sharp or blunt round, which showed in the RsB, RsI, RsL and RsW populations (Figure 2A, B; Figure 5K, L, N, Q). The type II tail is conoid, then sub-cylindrical, tail terminus round which showed in the RsP and RsA population (Figure 2C, D; Figure 5J). The type III tail is conoid with a fingerlike terminus which showed in the RsB, RsW and RsC populations (Figure 2E–H). The type IV tail is conoid with forked ends showed in the RsA population (Figure 2I). Among these, type I and II tail shapes were more frequent than the other two. In addition, tail shapes were not identical within the same population. The RsA population showed types II and IV, and the RsW population showed types I and II. The RsB, RsN and RsV populations showed types I, II and III, whereas the other populations showed most tail shapes as types I and II.

Males. The shortest individual male (572.5 µm) was found in the population from *Anthurium andraeanum* coded as RsJ, and the shortest males with average length of 602.2 µm were from the same host population but RsW. The longest male (770 µm) was found in the RsK population from *Calathea zebrina*, and the longest males with average length of 676.9 µm were found in the RsH population from *Philodendron* sp. The longest spicule (20.8 µm) was found in the RsY population, and the shortest (17.2 µm) in the RsJ population. Tail shape varied in and among populations: RsA, RsJ and RsW populations had type I and type III tails (Figure 2P). The RsB, RsI, RsL and RsT populations had type II and type III tails (Figure 2O). The RsXJ population had type I, II and III tails (Figure 2Q). The RsK, RsG, RsP and RsV had type I, II and III tails. The RsN population had type I (Figure 2M), II (Figure 2N) and III tails (Figure 2R). The remaining populations had type I and type II tails.

**Morphological observations of progeny of single females.** Females. The shortest female was found in the RsEs population (545 µm), and the shortest females with average length of 651.6 µm were found in the RsDs population. The longest individual female and longest females were found in the RsAs population (body length = 798.8 µm, the average body length = 743.4 µm, respectively). The head diameter and height varied from 9×4 µm–9.6×4.1 µm, and the stylet length varied from 18.3 μm to 19.4 μm. The head annuli varied in and among the populations. Two head annuli were found in the RsXJs population, 3 in the RsAs, RsEs, RsHs, RsIs, RsKs, RsLs, RsTs, RsKs and RsYs populations, 4 in the RsBs, RsDs, RsGs, RsJs, RsNs and RsPs populations, and 3–4 in the RsCs, RsMs and RsVs populations. The longest tails of female with average length of 85.3 μm were from the RsAs population, and shortest tails (68.8 μm) were from the RsDs population. The highest number of tail annuli (60) was from the RsPs population, and the least (24) was from the RsXJs population. Tail shape varied also.The tail type I predominated in the RsBs, RsCs, RsEs, RsLs, RsMs, RsPs, RsSs and RsYs populations (Figure 5I). Tail type I and II were found in the RsGs and RsTs population. Tail type I and III were found in the RsJs population (Figure 5O). Tail type I and III were found in the RsWs population (Figure 5G). Tail types II and III were found in the RsGs and RsTs population. Tail types I, II and III were each found in the RsAs, RsIs, RsNs and RsVs populations. Tail types II, III and IV were found in the RsHs populations (Figure 2J). And tail types I, II and III were found in the RsKs population.

Males. The shortest male was found in the RsHs population (527.5 µm), and the shortest males with average length of 594.8 µm were found in the RsDs populations. The longest male and longest males were both from the RsAs population (body length = 763.8 µm, the average length = 708.5 µm, respectively). The longest spicule was found in the RsLs and RsYs populations (20.5 µm), and the shortest spicule was found in the RsKs and RsWs population (18.1 µm). The tail shape varied also. In the RsJs and RsXJs population, the tail type was I. In the RsAs and RsSs population, the tail type was I and II; in the RsKs population, the tail types were I and III. In the RsMs and RsPs populations, the tail types were II and III. In the remaining populations, tail tail types were I, II and III.

**Scanning electron microscopy observation.** Nematodes progeny of 30 females.

**Figure 3.**
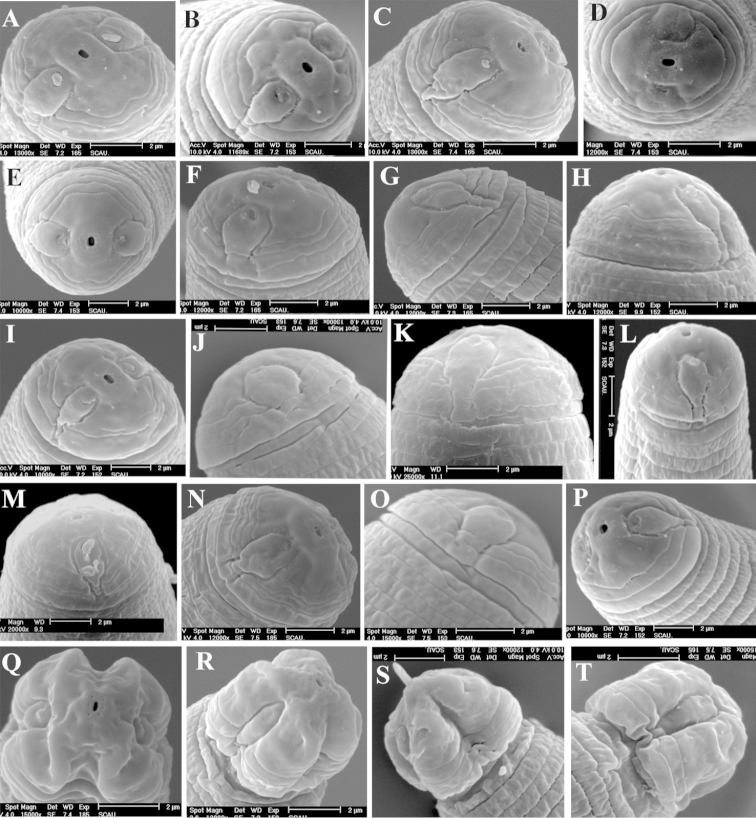
Lip region morphology of female and male of *Radopholus similis* Female: **A** Face view of RsA **B** Face view of RsC **C** Face view of RsD **D** Face view of RsT **E** Face view of RsT **F** Lateral view of RsE **G** Lateral view of RsE **H** Lateral view of RsK **I** Lateral view of RsXJ **J** Lateral view of RsT **K** Lateral view of RsP **L** Lateral view of RsL **M** Lateral view of RsY **N** Lateral view of RsEs **O** Lateral view of RsT **P** Lateral view of RsXJ. Male: **Q** Face view of RsTs **R** Lateral view of RsY **S** Lateral view of RsV **T** Lateral view of RsN.

**Figure 4.**
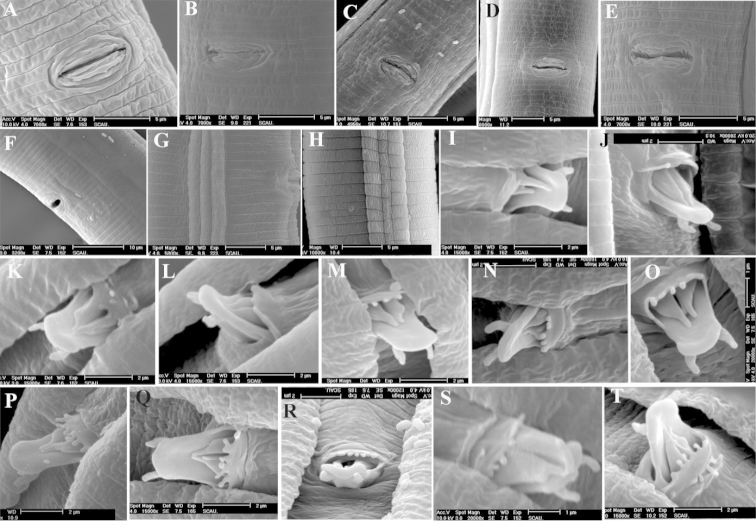
Annuli terminated at vulvar, incisures and genital papillae in cloacal region of *Radopholus similis* Annuli terminated at vulvar region of females: **A** One annulus in RsC **B** One and two annuli on each side in RsN **C** One and three annuli on each side in RxXJs **D** Three annuli in RsP **E** Two annuli in RsK **F** Four annuli in RsW. Incisures in lateral region of femlaes **G** RsH **H** RsJs. Number of genital papillae in cloacal region of males: **I** 0 genital papillae of RsEs **J** 1 genital papillae of RsKs. 2 genital papillae of RsP **L** 3 genital papillae of RsC **M** 4 genital papillae of RsB **N** 5 genital papillae of RsTs **O** 5 genital papillae of RsI **P** 6 genital papillae of RsB **Q** 7 genital papillae of RsM **R** 8 genital papillae of RsG **S** 8 genital papillae in double row of RsD **T** 9 genital papillae in double row of RsP.

Females. The main differences in morphological characters of females observed by SEM were shape of labial disc, terminal position of lateral lip and annuli terminated at vulva. The shape of the labial disc of all the 20 populations was divided into three types: hexagonal (RsC) (Figure 3B), with the two dorsal lip and ventral lip obviously not fused; round-elongate, due to the fusion of the two dorsal lips and the ventral lips, respectively (RsD, RsG, RsH and RsL populations) (Figure 3D); and sub-hexagonal, because of the two dorsal lips and two ventral lips partially fused, with a depression formed between the two dorsal lips and two ventral lips (all the other populations) (Figure 3A, E). The lateral lips terminated differently depending on the population. In the RsS and RsXJ populations, the lateral lips appeared to terminate at the end of second head annulus (Figure 3I, P). In the RsA, RsG, RsV and RsW populations, the lateral lips terminated before the third annulus (Figure 3M). In the RsB, RsC, RsD, RsH, RsJ, RsM and RsN populations, the lateral lips extended to the end of the third head annulus (Figure 3C, G). In the RsI, RsK, RsP and RsY populations, the lateral lips terminated at the end of the fourth head annulus (Figure 3H, K). In the RsL population, the lateral lips terminated over the end of the last annulus (Figure 3L). In the RsE population, one side of the lateral lips terminated at the end of the third annulus, and the other side of lateral lips terminated in middle of the second head annulus (Figure 3F). In the RsT population, one side of the lateral lips terminated before the third annulus, and the other side of lateral lips terminated at the end of third annulus (Figure 3J, O). The annuli terminated differently in the vulval area also varied in and among the populations. Among them, one annulus terminated at the vulva were found in the RsC and RsM populations (Figure 4A), two annuli in the RsA, RsB, RsD, RsG, RsE, RsH, RsI, RsJ, RsK, RsL, RsT, and RsV populations (Figure 4E), and three annuli in the RsP and RsS populations (Figure 4D). In addition, two or four annuli terminated at the vulva were found in the RsW population (Figure 4F). Whereas one on one side and two on the other side were found in the RsN, RsY and RsXj populations (Figure 4B).

**Figure 5.**
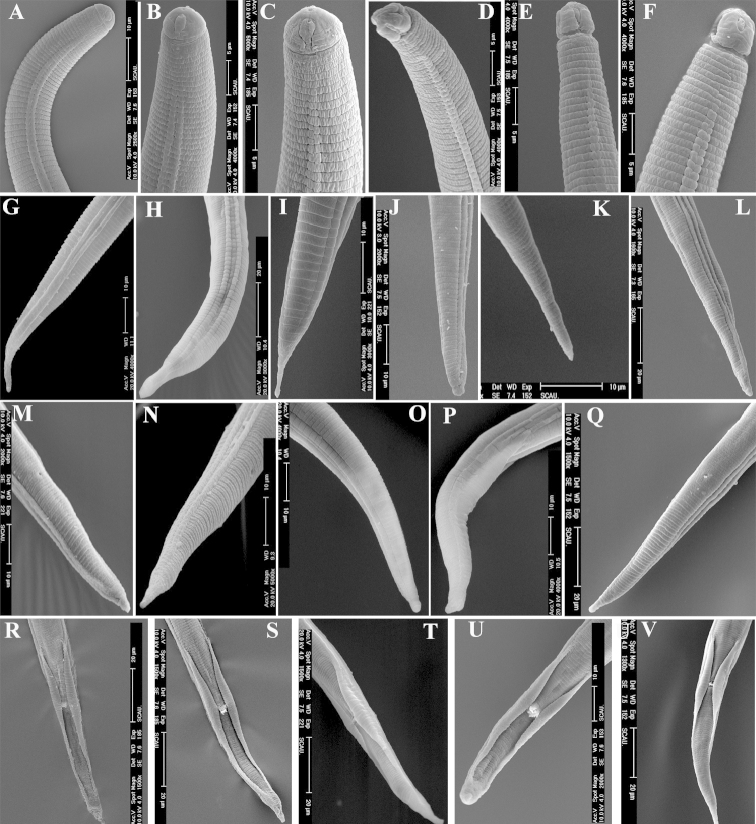
Anterior region and tail of *Radopholus similis* Anterior region. **A** Female RsTs **B** Female RsL **C** Female RsLs **D** Male RsV **E** Male RsXJ **F** Male RsAs Female tails **G** Female type I of RsWs **H** RsXJs **I** RsCs **J** RsP **K** RsW **L** RsI **M** RsXJs **N** RsW **O** RsJs **P** RsXJs **Q** RsL. Male tails: **R** RsG **S** RsLs **T** RsHs **U** RsVs **V** RsS.

Males. The main differences in morphological characters of males observed by SEM were in head shape and number of genital papillae on the anterior cloacal apertures. The head region was four-lobed, formed by a longitudinal constriction, and the first annuli were wider than the remaining head annuli (Figure 3Q–T). Three annuli were found in the RsA, RsI, RsJ, RsK and RsN populations (Figure 3T), and four annuli in the remaining populations (Figure 3R). The number of genital papillae on the anterior cloacal apertures varied in and among populations, usually 0–9 in a single row (Figure 4I–R), but in the RsD and RsP populations, the genital papillae were arranged in a double row (Figure 4S–T).

**Nematode progeny of the single females.** Observation of the progeny of the single female by SEM showed no obvious differences between the progeny of the 30 females and the single female inoculated on carrot discs, but some variations were found within the same population. Regarding the terminal position of lateral lips, only the RsBs, RsDs, RsMs and RsSs populations showed the same position with their counterpart of progeny of the 30 females, but the remaining populations did not. In the RsHs and RsXjs populations, the lateral lips terminated at the end of the second head annulus. In the RsCs population, the lateral lips terminated before the third head annulus. In the RsAs, RsEs, RsKs, RsTs and RsVs populations, the lateral lips terminated in the middle of the third head annulus. In the RsGs, RsIs, RsLs, RsWs and RsYs populations, the lateral lips terminated at the end of the third head annulus. In the RsJs, RsNs and RsPs populations, the lateral lips terminated at the end of the fourth head annulus. Populations RsAs, RsBs, RsCs, RsDs, RsFs, RsGs, RsIs, RsJs, RsLs, RsMs, RsTs, RsVs and RsYs had the same annuli termination at the vulva as their counterpart of progeny of the 30 females, whereas the remaining populations did not. They were one annulus in the RsHs, RsSs and RsWs populations, two in the RsNs RsPs and RsYs populations, one on one side and two on the other side in the RsEs and RsKs populations, and one on one side and three annuli on the other side in the RsXjs population. Regarding the number of genital papillae on the anterior cloacal apertures of males, the same variations were observed within and among the populations with the progeny of the 30 nematodes.

**Morphological comparison between single female progeny and 30 females progeny.** There were no obvious morphological differences between single female progeny and corresponding 30 female progeny. Some variations within some measurements were noted but overlap of morphological measurements existed. Among them, in all populations except the RsM population, the average female body length of progeny of single female was shorter than that of 30 females from the same population. However, the average male body lengths were similar. Tail type varied within and among the same populations from progeny of single female and 30 females, and only the female tail type of the RsTs, RsNs and RsVs populations and the male tail type of the RsVs populations were identical with the corresponding 30 females from the same population. All other populations showed divergence but with morphological character overlap.

**Figure 6.**
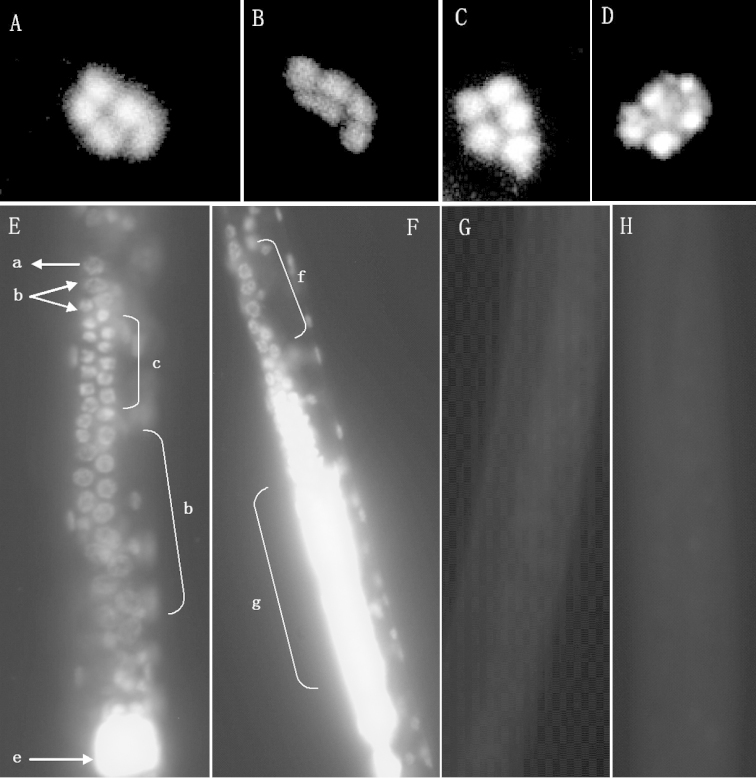
Haploid chromosomes and genital cells of *Radopholus similis* stained with DAPI Haploid chromosomes. **A** RsB population **B** RsL population **C** RsN population **D** RsY population; Genital cells **E** Female stained with DAPI **F** Male stained with DAPI **G** Female Non-stained with DAPI **H** Male non-stained with DAPI Arrows Arrow a: Cap cell; Arrow b: Somatic cells; Arrow c: Germinal zone; Arrow d: growth zone; Arrow e: spermatheca; Arrow f: testis; Arrow g: seminal vesicle.

**Karyotype analysis.** Staining burrowing nematode eggs at the single cell stage with DAPI enabled counting of chromosomes in polar bodies, and the result showed that all the twenty populations of *Radopholus similis* have the same haploid karyotype n = 5 (Figure 6A–D).

Specimens that had not been stained with DAPI were also examined to ensure that we were not observing auto-fluorescence. After staining adult nematodes of *Radopholus similis*, strong fluorescence in the spermatheca and testis was detected in females and males. Highly condensed chromosomes in meiosis were detected in genital ovaries (Figure 6D–H). We also observed the female reproductive system to be didelphic and the ovotestes to have extended glands, which were made up with ovary, oviduct, spermatozoa and uterus. A cap cell and three somatic cells were found at the tip of each ovary, and germina zone showed strong fluorescence because of highly condensed nucleic acids. The next growth zone showed the cytoplasm of single cells because of less condensed nucleic acid (Figure 6E).

## Discussion

### Morphological variations within different populations

All the morphological characters of *Radopholus similis* populations in this study were similar to those described by Huettel et al. (1986), Koshy et al. (1991) and Elbadri et al. (1999a, 1999b), even though there were some variations of morphological characters and measurements in and among populations. Our results showed that the lateral field structure and all morphometric values were almost stable. The main morphological diversity was manifested in number of female head annuli, shapes of female labial disc, terminal position of female lateral lips, number of annuli terminating at the vulval area, number of genital papillae before the male cloacal aperture, and tail shapes of females and males.

Elbadri et al. (1999a) analyzed the morphological characters in and among ten banana populations of *Radopholus similis* from Africa. The number of head annuli varied between 2–5. The labial disc was round or flat round. The lateral lip was terminated before the second or third head annulus, or at the end of the fourth or fifth head annulus. In addition, the lateral lips of the Ugandan and South African populations terminated at the different positions on both sides of the body. The annuli terminated at the vulva varied between 2–3 and the number of genital papillae on the anterior cloacal apertures varied between 0–8. Elbadri et al. (1999b) also compared the morphological characters in and among eight populations of *Radopholus similis* extracted from different hosts (banana, pepper, citrus and ornamental plants) from different continents (Asia, the Americas, Europe and Oceania), and found that in the banana and pepper populations, the number of head annuli varied between 2–4 and the shape of labial disc varied from hexagonal, subhexagonal and flat round. In the ornamental populations, the number of head annuli was 3, and the shape of labial disc was hexagonal. The lateral lips terminated at the end of the third annulus, or in the middle of the second or third annuli in all these populations. The number of annuli terminated at the vulva area varied from 1–3 on both sides, and in some nematodes. the number of annuli terminated at the vulva was different on both sides of the vulva. In addition, the number of genital papillae varied between 0–7 in and among populations.

Our study showed that the shape of female labial discs was hexagonal, sub-hexagonal and round-elongate. The number of female head annuli varied from 2–4. The terminated position of female lateral lips showed different situations which varied in and among populations. The number of genital papillae before male cloacal aperture varied from 0–9 in and among populations. What is interesting is that the genital papillae were arranged in double rows in two of the ornamental populations, RsP population from *Calathea zebrina* and RsD from *Chamaedorea cataractarum*, and this number was 9 and 8 respectively. The tail shape varied the most, was usually conoid, widely cylindrical or bearing a pointed end, and only one intercepted RsA population from *Calathea zebrina* showed forked ends. All 20 populations showed much more variations in tail shape than as described by Huettel et al. (1986) and Elbadri et al. (1999a, 1999b). Although the differences of morphological characters in and among various populations existed, these differences exist not only among the populations but also exist between the individuals within the same populations, so based on these morphological characters, we cannot separate different geographic or host populations of *Radopholus similis*.

Huettel and Yaegashi (1988) treated two physiological races of *Radopholus similis* *sensu lato* as two independent species, *Radopholus similis* *sensu stricto* (not attacking citrus) and *Radopholus citrophilus* (attacking citrus) according to the four different ultrastructures observed by SEM. They separated *Radopholus similis* *sensu stricto* from *Radopholus citrophilus* by the former having a hexagonal labial disc, the lateral lips terminated at the end of the third annulus, the annuli terminated at the vulva being 2, the number of genital papillae of male cloacal aperture varying between 0–2, and the latter having a round labial disc, the lateral lips terminated at the end of the third annulus, the annuli terminated at the vulva numbering 3, and the number of genital papillae of male cloacal aperture varied between 3–7. However, Koshy et al. (1991), Elbadri et al. (1998), Valette et al. (1998) and Elbadri et al. (1999a, 1999b) studied more populations of *Radopholus similis* *sensu lato*, and demonstrated that the four specific morphological characters between *Radopholus similis* *sensu stricto* and *Radopholus citrophilus* described by Huettel and Yaegashi (1988) showed considerable overlap, and they also treated *Radopholus citrophilus* as a synonym of *Radopholus similis*. Our results also show that the four morphological characters of the 20 populations showed considerable overlap in and among populations, even between the progeny of single females and that of 30 females from the same population. In addition, all 20 populations showed other morphological divergences, whether in morphometric values or in morphological characters. Even most of morphometric values and characters showed some variation in the progeny of the single females. The ultrastructure of nematodes from the progeny of single females also showed some variations compared to their corresponding progeny of 30 females. Therefore, according to the our and reported morphological characters, we cannot separate *Radopholus citrophilus* as a separate species, and we cannot even separate different populations of *Radopholus similis*.

**Karyotype variations analysis of *Radopholus similis*.** Huettel and Dickson (1981a) and Huettel et al. 1984a) reported the chromosome numbers of banana and citrus races of *Radopholus similis* as n = 4 and n = 5, respectively. Huettel et al. (1984a) reported the karyotype of three ornamental plant populations, and the karyotype from *Philodendron* sp. and *Calathea* sp. populations as n = 4, while that of *Anthurium* was n = 5; therefore, they proposed that citrus race can be distinguished from banana race based on the haploid number of chromosomes. Hahn et al. (1996a) and Kaplan and Opperman (2000) reported that the chromosome number of banana races of *Radopholus similis* was 5. In this study, among the 20 populations, including two *Philodendron*, six *Calathea* sp. and five *Anthurium* populations, the results showed that the haploid chromosome number of all the populations was 5. Therefore, our results confirm previous studies, and we can conclude that it is impossible to separate different races of *Radopholus similis* according to karyotype.

## Conclusion

According to the morphological characters and karyotype of the 20 populations of *Radopholus similis*, a diversity of morphological characters of *Radopholus similis* exists in and among the populations. According to our and previously reported results, we also suggestthe banana race and citrus race of *Radopholus similis* cannot be separated, and *Radopholus citrophilus* cannot be accepted as a sibling species by optical or SEM morphological values and characters or by karyotype.
